# Adverse Events of Interest Following Influenza Vaccination in the First Season of Adjuvanted Trivalent Immunization: Retrospective Cohort Study

**DOI:** 10.2196/25803

**Published:** 2022-03-28

**Authors:** Simon de Lusignan, Ruby S M Tsang, Oluwafunmi Akinyemi, Jamie Lopez Bernal, Gayatri Amirthalingam, Julian Sherlock, Gillian Smith, Maria Zambon, Gary Howsam, Mark Joy

**Affiliations:** 1 University of Oxford Oxford United Kingdom; 2 Public Health England London United Kingdom; 3 Royal College of General Practitioners London United Kingdom

**Keywords:** influenza, influenza vaccines, adverse events of interest, computerized medical record systems, sentinel surveillance

## Abstract

**Background:**

Vaccination is the most effective form of prevention of seasonal influenza; the United Kingdom has a national influenza vaccination program to cover targeted population groups. Influenza vaccines are known to be associated with some common minor adverse events of interest (AEIs), but it is not known if the adjuvanted trivalent influenza vaccine (aTIV), first offered in the 2018/2019 season, would be associated with more AEIs than other types of vaccines.

**Objective:**

We aim to compare the incidence of AEIs associated with different types of seasonal influenza vaccines offered in the 2018/2019 season.

**Methods:**

We carried out a retrospective cohort study using computerized medical record data from the Royal College of General Practitioners Research and Surveillance Centre sentinel network database. We extracted data on vaccine exposure and consultations for European Medicines Agency–specified AEIs for the 2018/2019 influenza season. We used a self-controlled case series design; computed relative incidence (RI) of AEIs following vaccination; and compared the incidence of AEIs associated with aTIV, the quadrivalent influenza vaccine, and the live attenuated influenza vaccine. We also compared the incidence of AEIs for vaccinations that took place in a practice with those that took place elsewhere.

**Results:**

A total of 1,024,160 individuals received a seasonal influenza vaccine, of which 165,723 individuals reported a total of 283,355 compatible symptoms in the 2018/2019 season. Most AEIs occurred within 7 days following vaccination, with a seasonal effect observed. Using aTIV as the reference group, the quadrivalent influenza vaccine was associated with a higher incidence of AEIs (RI 1.46, 95% CI 1.41-1.52), whereas the live attenuated influenza vaccine was associated with a lower incidence of AEIs (RI 0.79, 95% CI 0.73-0.83). No effect of vaccination setting on the incidence of AEIs was observed.

**Conclusions:**

Routine sentinel network data offer an opportunity to make comparisons between safety profiles of different vaccines. Evidence that supports the safety of newer types of vaccines may be reassuring for patients and could help improve uptake in the future.

## Introduction

Seasonal epidemics of influenza lead to an estimated 3 to 5 million cases of severe illness and about 290,000 to 650,000 respiratory deaths annually worldwide, and vaccination is the most effective form of influenza prevention [1]. The United Kingdom has a long-standing national influenza vaccination program for targeted population groups, and different types of vaccines are recommended for these groups to achieve optimal immunogenicity and effectiveness. In the 2018/2019 season, following updated guidance from the Joint Committee on Vaccination and Immunisation, the adjuvanted trivalent influenza vaccine (aTIV) was recommended for adults 65 years and older, the quadrivalent influenza vaccine (QIV) for adults aged 18 years to younger than 65 years in clinical at-risk groups, and the quadrivalent live attenuated influenza vaccine (LAIV) for children.

Seasonal influenza vaccines are known to be associated with a range of adverse events of interest (AEIs), including minor ones like fever, malaise, and injection site soreness, and more serious AEIs such as anaphylaxis have been documented [2]. Study findings show an association between seasonal flu vaccination and Guillain-Barré syndrome in some years [3], while oculorespiratory syndrome is rarely reported [4]. Since 2016, the European Medicines Agency (EMA) required all vaccine manufacturers to conduct annual postmarketing enhanced safety surveillance studies. As 2018/2019 is the first influenza season following the licensure of aTIV in the United Kingdom, it is yet unknown whether this particular type of vaccine is associated with a higher incidence of AEIs compared to other types of vaccines.

We conducted this study to calculate the relative incidence (RI) of AEIs following seasonal influenza vaccination; compare the incidence of AEIs between aTIV, QIV, and LAIV; and explore whether the settings in which the vaccination took place had an effect on AEIs.

## Methods

### Study Design and Data Sources

We used a retrospective cohort design involving computerized medical record data from the Royal College of General Practitioners (RCGP) Research and Surveillance Centre (RSC) sentinel network. The RCGP RSC is a nationally representative primary care sentinel network with over 50 years of history in influenza and respiratory disease surveillance [5]. At the time of this study, the RCGP RSC network contained more than 8 million patient records from 336 member general practices across England. Clinical encounters of patients visiting their general practitioners (GPs) for adverse events are recorded into a GP electronic medical record system using 5–byte Read or Clinical Terms Version 3 codes.

We extracted data from all patients who had received a seasonal influenza vaccine between September 1, 2018, and April 30, 2019. We excluded patients older than 100 years, those who attended practices involved in EMA enhanced surveillance programs [6-8], and those who received monovalent pandemic influenza vaccines. Patients were followed up retrospectively for occurrence of a list of AEIs prespecified by the EMA, and those who presented with compatible symptoms were included in this study [9].

### Variables

We extracted the following data: age, sex, self-reported ethnicity, index of multiple deprivation (IMD), vaccination date, vaccine manufacturer, vaccine valency (number of viral strains included), route of administration, date of any AEI, type of AEI, and dates of registration and deregistration at the practice.

### Determination of Vaccine Type and Vaccination Setting

First, we used prescription data (where available) and clinical event data to assign drug name, manufacturer, valency, and route of administration. Second, for the records without prescription data, batch numbers were collated for each drug name to assign manufacturer, valency, and administration route. For records with both prescription and clinical data available, conflicting data were excluded from the analysis. We coded vaccinations where we had sufficient information to identify the vaccination as having taken place within a practice.

### Statistical Analyses

We used a self-controlled case series approach [10,11], a method typically used to investigate adverse events following administration of medications or vaccinations. It is a case-only method for investigating the association between a time-varying exposure and an outcome event, in which any time-invariant confounding is automatically controlled for as each patient acts as their own control.

We computed descriptive statistics to provide an overview of the demographic characteristics of the study sample.

**Figure 1:**
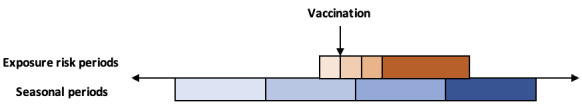
Simplified illustration of SCCS model for a hypothetical individual showing four risk periods (days –7 to –1, days 0 to 6, days 7 to 13, and days 14 to 45, where day 0 is day of vaccination), and four of eight 30-day periods in the influenza season.

We conducted three separate models to address the three aforementioned study aims. The observation period used in all models was from September 1, 2018, to April 30, 2019. Where an individual registered with the practice after September 1, 2018, or deregistered with the practice before April 30, 2019, their observation period was defined as the number of days they remained registered. In model 1, we defined the exposure risk periods relative to the day of vaccination (day 0) as days –7 to –1, days 0 to 6, days 7 to 13, and days 14 to 45, and defined seasonal periods within the observation period as days 0 to 29, days 30 to 59, days 60 to 89, days 90 to 119, days 120 to 149, days 150 to 179, days 180 to 209, and days 210 to 241 from the beginning of the influenza season (see Figure 1 for an illustration). The time outside of the defined exposure risk periods is used as the control for each individual patient. Where an individual received the vaccination very early or very late in the season, parts of the exposure risk periods that fell outside of our defined observation period were not included. We calculated the RI of AEIs following vaccination for the different exposure periods and for the different seasonal periods. In models 2 and 3, we focused on the first 7 days post vaccination as the exposure risk period and modeled potential modification effects of vaccine type and vaccination settings by including an interaction term in the model [12].

All statistical analysis was performed using R 3.4.4 (R Foundation for Statistical Computing) [13] with the packages tidyverse version 1.2.1 [14], SCCS version 1.1 [15], lubridate [16], and tableone [17]. Graphical output was generated using the packages ggplot2 [18] and ggthemes version 3.5.0 [19].

### Ethical Considerations

All potentially identifiable data were pseudonymized as close to the source as possible and not made available to researchers; data were not extracted for patients who opted out of data sharing. All data are stored and processed at the RCGP RSC secure data and analytics hub, the University of Surrey. According to the Health Research Authority and Medical Research Council Regulatory Support Centre’s online decision tool, this study falls under the category of service evaluation and does not require further ethical review. This study was approved by RCGP.

## Results

### Study Participants

**Figure 2:**
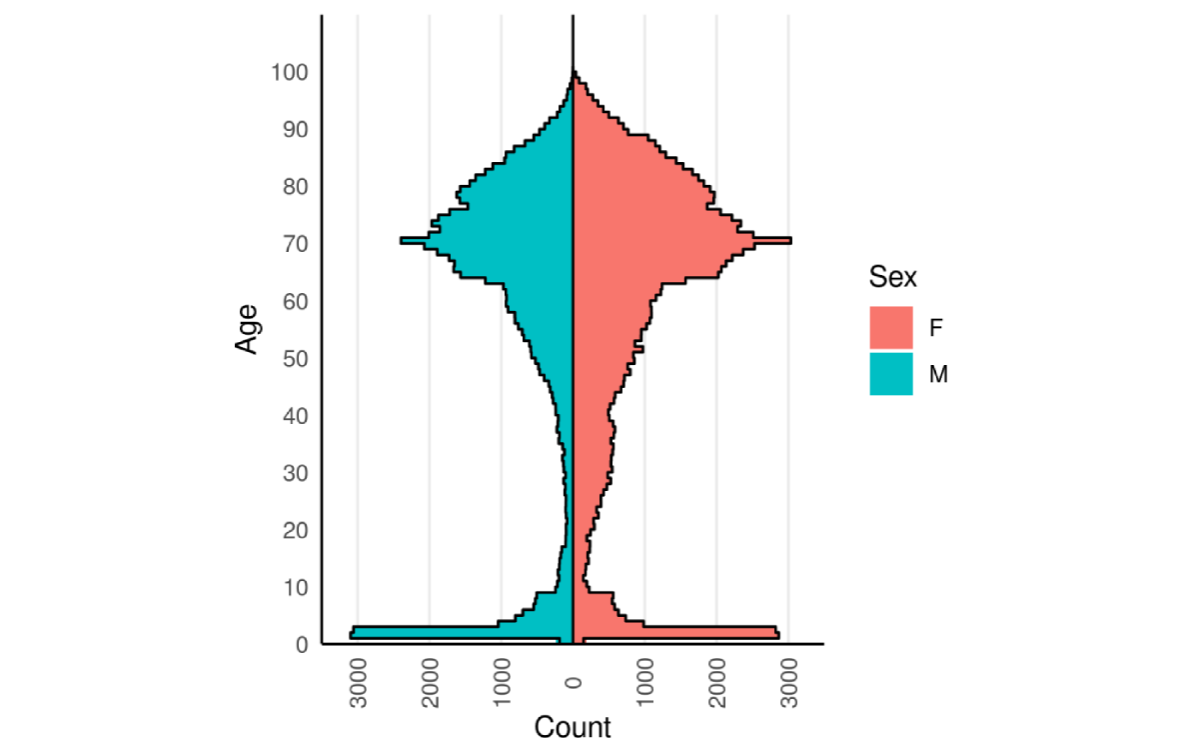
Age-sex profile for seasonal influenza vaccine recipients in the United Kingdom’s Royal College of General Practitioners Research and Surveillance Centre network who reported adverse events of interest between September 1, 2018, and April 30, 2019 (n=165,723). F: female; M: male.

A total of 1,024,160 unique individuals who received seasonal influenza vaccinations were identified, of which 165,723 individuals presented with symptoms compatible with vaccine-related AEIs. Baseline demographic characteristics of the study participants are presented in Table 1. The median age of the cohort was 66 years, with slightly more women than men, and the majority of the study participants were of White ethnicity. The age-sex profile showed a peak between the ages of 2 and 9 years, and a marked increase in uptake from the age of 65 years (Figure 2). The IMD distribution, however, showed a slight overrepresentation of patients from less deprived neighborhoods (see Table 1).

**Table 1:** Demographic characteristics of seasonal influenza vaccine recipients in the United Kingdom’s Royal College of General Practitioner Research and Surveillance Centre network who reported adverse events of interest between September 1, 2018, and April 30, 2019, (n=165,723) by type of vaccine.

		aTIV^a^ (n=92,336)	QIV^b^ (n=51,616)	LAIV^c^ (n=21,771)
Age (years), mean (SD)		76.27 (8.2)	47.58 (14.8)	4.86 (4.0)
**Sex, n (%)**				
	Female	52,598 (57.0)	32,702 (63.4)	10,596 (48.7)
	Male	39,738 (43.0)	18,914 (36.6)	11,175 (51.3)
**Ethnicity, n (%)**				
	White	71,358 (77.3)	36,206 (70.1)	11,677 (53.6)
	Asian	3104 (3.4)	4849 (9.4)	1595 (7.3)
	Black	869 (0.9)	1774 (3.4)	493 (2.3)
	Mixed	224 (0.2)	548 (1.1)	525 (2.4)
	Other	313 (0.3)	620 (1.2)	220 (1.0)
	Missing data	16,468 (17.8)	7619 (14.8)	7261 (33.4)
**Index of multiple deprivation quintile, n (%)**				
	1 (most deprived)	11,143 (12.1)	11,665 (22.6)	3690 (16.9)
	2	12,816 (13.9)	9534 (18.5)	3609 (16.6)
	3	18,685 (20.2)	9428 (18.3)	3923 (18.0)
	4	22,894 (24.8)	10,207 (19.8)	4712 (21.6)
	5 (least deprived)	25,369 (27.5)	9726 (18.8)	5339 (24.5)
	Missing data	1429 (1.5)	1056 (2.0)	498 (2.3)
BMI, mean (SD)		28.06 (5.9)	30.52 (7.8)	18.64 (5.2)
**Smoking status, n (%)**				
	Nonsmoker	47,068 (51.0)	27,406 (53.1)	2991 (13.7)
	Ex-smoker	36,032 (39.0)	12,614 (24.4)	36 (0.2)
	Active smoker	8414 (9.1)	10,011 (19.4)	111 (0.5)
	Missing data	822 (0.9)	1585 (3.1)	18,633 (85.6)
**Alcohol consumption level, n (%)**				
	Nondrinker	8558 (9.3)	5544 (10.7)	164 (0.8)
	Safe	9172 (9.9)	4982 (9.7)	41 (0.2)
	Hazardous	14,649 (15.9)	6307 (12.2)	40 (0.2)
	Alcoholism	1463 (1.6)	1654 (3.2)	1 (0.0)
	Missing data	58,494 (63.3)	33,129 (64.2)	21,525 (98.9)

^a^aTIV: adjuvanted trivalent influenza vaccine.

^b^QIV: quadrivalent influenza vaccine.

^c^LAIV: live attenuated influenza vaccine.

### Vaccine Type

We were able to identify the vaccine administered in 77.8% (797,285/1,024,160) of the records. The main types of vaccines used in 2018/2019 included aTIV, QIV, and LAIV. A small number of trivalent vaccines (TIVs) were also identified (n=1526); as they were not recommended in the national influenza vaccination program, we excluded them from the analyses.

### Adverse Event of Interest

The incidence rates of AEIs for which patients sought consultation in the 7 days post vaccination are listed in Table 2, grouped by category of surveillance condition. We observed AEIs in every EMA category, from the most common ones of cough, myalgia, rash, and headache to the more severe ones such as Guillain-Barré syndrome (n=4) and anaphylaxis (n=6).

**Table 2:** Number of adverse events of interest reported following seasonal influenza vaccination in the United Kingdom’s Royal College of General Practitioners Research and Surveillance Centre between September 1, 2018, and April 30, 2019, by type of vaccine.

		aTIV^a^ (total doses n=454,567)				QIV^b^ (total doses n=238,654)				LAIV^c^ (total doses n=102,538)				
		Total events in season, n	Events within 7 days of vaccination, n	7-day cumulative incidence (events per 100,000 doses)	Events within 7 days/total events in season	Total events in season, n	Events within 7 days of vaccination, n	7-day cumulative incidence (events per 100,000 doses)	Events within 7 days/total events in season	Total events in season, n	Events within 7 days of vaccination, n	7-day cumulative incidence (events per 100,000 doses)	Events within 7 days/total events in season	
**Fever/pyrexia**														
	Fever (unspecified)	2413	75	16.50	0.03	1959	83	34.78	0.04	3107	87	84.85	0.03	
	Mild fever (≤38.5 °C)	6150	133	29.26	0.02	3493	90	37.71	0.03	5248	140	136.53	0.03	
	Moderate fever (38.6-39.5 °C)	563	13	2.86	0.02	344	13	5.45	0.04	1352	23	22.43	0.02	
	High fever (>39.5 °C)	67	0	0	0	28	1	0.42	0.04	220	1	0.98	0.00	
**Gastrointestinal**														
	Decreased appetite	1284	71	15.62	0.06	487	36	15.08	0.07	359	14	13.65	0.04	
	Diarrhea	9388	350	77.00	0.04	3631	185	77.52	0.05	1262	32	31.21	0.03	
	Nausea	2365	110	24.20	0.05	1170	82	34.36	0.07	166	14	13.65	0.08	
	Vomiting	2323	61	13.42	0.03	1571	69	28.91	0.04	1264	39	38.03	0.03	
**General nonspecific symptoms**														
	Drowsiness	331	10	2.20	0.03	70	4	1.68	0.06	17	0	0	0	
	Fatigue	5406	290	63.80	0.05	3449	290	121.51	0.08	297	11	10.73	0.04	
	Headache	7169	356	78.32	0.05	8883	617	258.53	0.07	919	34	33.16	0.04	
	Irritability	58	9	1.98	0.16	81	4	1.68	0.05	17	1	0.98	0.06	
	Malaise	4451	144	31.68	0.03	1596	65	27.24	0.04	367	11	10.73	0.03	
Local symptoms (ie, local erythema)		49	5	1.10	0.10	29	10	4.19	0.34	6	0	0	0	
**Musculoskeletal**														
	Arthropathy	515	28	6.16	0.05	321	23	9.64	0.07	3	0	0	0	
	Muscle aches/myalgia	35,421	1911	420.40	0.05	17,836	1381	578.66	0.08	951	32	31.21	0.03	
**Neurological**														
	Bell palsy	217	9	1.98	0.04	193	9	3.77	0.05	12	0	0	0	
	Guillain-Barré syndrome	26	3	0.66	0.12	4	0	0	0	1	0	0	0	
	Peripheral tremor	1379	79	17.38	0.06	385	27	11.31	0.07	22	2	1.95	0.09	
	Seizure/febrile convulsions	784	33	7.26	0.04	693	21	8.80	0.03	263	9	8.78	0.03	
Rash		13,351	671	147.61	0.05	5888	445	186.46	0.08	3920	145	141.41	0.04	
**Respiratory/miscellaneous**														
	Conjunctivitis	5140	269	59.18	0.05	1892	111	46.51	0.06	1709	57	55.59	0.03	
	Coryza	610	27	5.94	0.04	491	30	12.57	0.06	347	20	19.50	0.06	
	Cough	42,829	1882	414.02	0.04	21,251	1500	628.52	0.07	8408	348	339.39	0.04	
	Epistaxis	3142	114	25.08	0.04	790	52	21.79	0.07	586	26	25.36	0.04	
	Hoarseness	1168	49	10.78	0.04	619	37	15.50	0.06	37	0	0	0	
	Influenza-like illness	1283	64	14.08	0.05	1347	54	22.63	0.04	205	9	8.78	0.04	
	Nasal congestion	3041	149	32.78	0.05	1727	119	49.86	0.07	465	19	18.53	0.04	
	Oropharyngeal pain	3488	144	31.68	0.04	3926	177	74.17	0.05	1915	58	56.56	0.03	
	Rhinorrhea	654	29	6.38	0.04	301	25	10.48	0.08	243	6	5.85	0.02	
	Wheezing	2873	191	42.02	0.07	2633	370	155.04	0.14	2302	119	116.05	0.05	
**Sensitivity/anaphylaxis**														
	Anaphylactic reactions	32	0	0	0	69	4	1.68	0.06	15	0	0	0	
	Facial edema	280	8	1.76	0.03	187	8	3.35	0.04	20	0	0	0	
	Hypersensitivity reactions	750	48	10.56	0.06	697	68	28.49	0.10	289	14	13.65	0.05	

^a^aTIV: adjuvanted trivalent influenza vaccine.

^b^QIV: quadrivalent influenza vaccine.

^c^LAIV: live attenuated influenza vaccine.

### Timing of AEIs

We observed a slight reduction in the incidence of AEIs in the 7 days prior to vaccination (RI 0.91, 95% CI 0.89-0.94). The 7 days following vaccination showed an elevated incidence (RI 1.88, 95% CI 1.84-1.91). The period of day 8 to 14 was not associated with a significant increase in incidence, and the period of day 15 to 45 only showed a marginally increased incidence (RI 1.01, 95% CI 1.00-1.03; Table 3).

We also observed a seasonal pattern of AEIs, with increasing incidence as the influenza season progresses, reaching a peak around February, and then the incidence declines until the end of the season. The exception to this pattern is the 30-day period that encompasses the end-of-year holidays, which showed a lower incidence compared to the preceding and succeeding 30-day periods (Table 3).

**Table 3:** Model 1: relative incidence of adverse events of interest in the exposure risk and seasonal periods, as reported by seasonal influenza vaccine recipients in the United Kingdom’s Royal College of General Practitioners Research and Surveillance Centre network between September 1, 2018, and April 30, 2019.

		Relative incidence (95% CI)	*P* value
**Exposure risk period**			
	Days –7 to –1	0.91 (0.89-0.94)	<.001
	Days 0 to 6	1.88 (1.84-1.91)	<.001
	Days 7 to 13	1.01 (0.99-1.04)	.28
	Days 14 to 45	1.01 (1.00-1.03)	.03
**Time from start of influenza season (reference: days 0 to 29)**			
	Days 30 to 59	1.11 (1.09-1.13)	<.001
	Days 60 to 89	1.15 (1.14-1.17)	<.001
	Days 90 to 119	1.07 (1.06-1.09)	<.001
	Days 120 to 149	1.29 (1.27-1.31)	<.001
	Days 150 to 179	1.33 (1.31-1.35)	<.001
	Days 180 to 209	1.26 (1.24-1.28)	<.001
	Days 210 to 241	1.08 (1.06-1.10)	<.001

### Vaccine Type and Vaccination Setting

To compare the incidence of AEIs associated with the different types of vaccines, we incorporated an interaction term in the model. The results showed that relative to aTIV, QIV was associated with more AEIs (RI 1.46, 95% CI 1.41-1.52), whereas LAIV was associated with fewer AEIs (RI 0.78, 95% CI 0.73-0.83; Table 4).

Similarly, we added an interaction term to the model to explore whether vaccination setting had an impact on rates of AEIs. There appeared to be no significant difference in rates of AEIs whether or not the vaccination took place within a practice (RI 0.97, 95% CI 0.66-1.43; Table 5).

**Table 4:** Model 2: relative incidence of adverse events of interest, as reported by seasonal influenza vaccine recipients in the United Kingdom’s Royal College of General Practitioners Research and Surveillance Centre network between September 1, 2018, and April 30, 2019, and modification effects of vaccine type.

		Relative incidence (95% CI)	*P* value
**Exposure risk period**			
	Days 0 to 6	1.61 (1.57-1.65)	<.001
**Time from start of influenza season (reference: days 0 to 29)**			
	Days 30 to 59	1.11 (1.09-1.12)	<.001
	Days 60 to 89	1.16 (1.15-1.18)	<.001
	Days 90 to 119	1.08 (1.07-1.10)	<.001
	Days 120 to 149	1.30 (1.28-1.32)	<.001
	Days 150 to 179	1.34 (1.32-1.36)	<.001
	Days 180 to 209	1.27 (1.25-1.29)	<.001
	Days 210 to 241	1.09 (1.07-1.10)	<.001
**Vaccine type (reference: adjuvanted trivalent influenza vaccine)**			
	Quadrivalent influenza vaccine	1.46 (1.41-1.52)	<.001
	Live attenuated influenza vaccine	0.78 (0.73-0.83)	<.001

**Table 5:** Model 3: relative incidence of adverse events of interest, as reported by seasonal influenza vaccine recipients in the United Kingdom’s Royal College of General Practitioners Research and Surveillance Centre network between September 1, 2018, and April 30, 2019, and interaction term for vaccination setting.

		Relative incidence (95% CI)	*P* value
**Exposure risk period**			
	Days 0 to 6	1.85 (1.25-2.73)	.002
**Time from start of influenza season (reference: days 0 to 29)**			
	Days 30 to 59	1.11 (1.09-1.13)	<.001
	Days 60 to 89	1.16 (1.14-1.18)	<.001
	Days 90 to 119	1.08 (1.06-1.10)	<.001
	Days 120 to 149	1.30 (1.28-1.32)	<.001
	Days 150 to 179	1.34 (1.32-1.36)	<.001
	Days 180 to 209	1.27 (1.25-1.29)	<.001
	Days 210 to 241	1.09 (1.07-1.10)	<.001
**Vaccination setting (reference: not in practice)**			
	In practice	0.97 (0.66-1.43)	.88

## Discussion

### Key Findings

In this study, we examined the incidence of AEIs following seasonal influenza vaccination, and the moderation effects of vaccination type and setting. We demonstrated that AEIs most occurred within 7 days post vaccination, although a small increase in incidence was also detected between days 15 and 45. Similar to observations from earlier influenza seasons, we also found a small “healthy vaccinee” effect in the 7 days leading up to vaccination, possibly due to patients who felt unwell deferring vaccination [20]. The seasonal pattern we observed was expected, as many of the AEIs are more common in winter.

Vaccination type had a significant effect on the incidence of AEIs in the 7 days post vaccination; we found that LAIV was associated with the lowest incidence, followed by aTIV and QIV. The incidence of AEIs following immunization with LAIV was 22% lower than that with aTIV, which in turn had a 46% lower incidence of AEIs than QIV. Data from earlier influenza seasons also showed a similar pattern where LAIV was associated with lower incidence of AEIs compared to QIV [20]. The finding that aTIV was associated with lower rates of AEIs than QIV was unexpected, as previous clinical trials and postlicensure studies have either reported higher reactogenicity with aTIV compared to TIV or similar safety profiles between the two [21-23].

Vaccination setting did not influence the incidence of AEIs; receiving the influenza vaccine outside of a practice was not associated with higher rates of AEIs. In the United Kingdom, most of these vaccinations not administered in a practice would have taken place in a pharmacy. In recent years, several countries have introduced pharmacy-based influenza vaccination services to enhance vaccination coverage. Their convenience and accessibility are thought to address some of the factors that contribute to vaccine hesitancy. Previous studies have reported that patient experiences are generally positive [24] and that vaccine delivery was safe [25]. Our findings are in accordance with prior research that influenza vaccines administered outside of a practice are safe, and leveraging this to enhance vaccination coverage may be particularly important in a pandemic, and commissioning other providers in addition to pharmacists may be an option to consider. The only downside of pharmacist vaccination is that there is no electronic transmission of administration into GP computerized medical record systems [26].

### Strengths and Limitations

The RCGP RSC is a nationally representative sentinel network, and practices receive feedback about data quality, so only data that meet a quality threshold are included in our report. However, it is important to acknowledge that the data used in this study is based on GP consultations, and patients are unlikely to seek medical attention for commonly expected or minor AEIs but tend to self-manage instead, or they may have directly attended the hospital for more severe AEIs. Furthermore, we were unable to identify brand-specific information for a proportion of the vaccinations; most often this is where vaccines are not administered in general practice [26]. Given that different types of vaccines were offered to different age groups in the 2018/2019 season, it is possible that the differences in rates of AEIs that we observed reflect differences in reactogenicity.

### Further Research

To date, most vaccines have had egg-based manufacturing, but in the 2019/2020 season cell-based manufactured vaccines were introduced [27]. A similar comparison of AEIs should be conducted to compare cell-based with egg-based QIV. Additionally, the sentinel system has had to adapt for the COVID-19 pandemic [28,29]; this has resulted in the expansion of the network size and the strengthening of its infrastructure [30]. The United Kingdom has ordered over 90 million COVID-19 vaccine doses [31], and the sentinel system could be used to monitor AEIs associated with its administration.

### Conclusions

The incidence of AEIs varied between different types of vaccines and can be compared using routine sentinel network data. Here, we report that aTIV are associated with fewer AEIs than QIV, but more AEIs than LAIV. Rates of AEIs were similar whether a vaccine took place in or outside of a practice. These findings should be reassuring for patients, address some of the factors that contribute to vaccine hesitancy, and may help improve vaccination coverage in future influenza seasons.

## Abbreviations

AEI: adverse event of interest
aTIV: adjuvanted trivalent influenza vaccine
EMA: European Medicines Agency
GP: general practitioner
IMD: index of multiple deprivation
LAIV: live attenuated influenza vaccine
QIV: quadrivalent influenza vaccine
RCGP: Royal College of General Practitioners
RI: relative incidence
RSC: Research and Surveillance Centre
TIV: trivalent vaccine
